# Brain iron deposits and lifespan cognitive ability

**DOI:** 10.1007/s11357-015-9837-2

**Published:** 2015-09-17

**Authors:** Maria del C. Valdés Hernández, Stuart Ritchie, Andreas Glatz, Mike Allerhand, Susana Muñoz Maniega, Alan J. Gow, Natalie A. Royle, Mark E. Bastin, John M. Starr, Ian J. Deary, Joanna M. Wardlaw

**Affiliations:** 1 Department of Neuroimaging Sciences, Centre for Clinical Brain Sciences, University of Edinburgh, 49 Little France Crescent, Chancellor’s Building, Edinburgh, EH16 4SB UK; 2 Department of Psychology, University of Edinburgh, Edinburgh, UK; 3 Department of Medical and Radiological Sciences, University of Edinburgh, Edinburgh, UK; 4 Department of Geriatric Medicine, University of Edinburgh, Edinburgh, UK; 5 Centre for Cognitive Ageing and Cognitive Epidemiology, University of Edinburgh, Edinburgh, UK; 6 Department of Psychology, School of Life Sciences, Heriot-Watt University, Edinburgh, UK

**Keywords:** Iron deposits, MRI, Ageing, Cognition, White matter hyperintensities

## Abstract

Several studies have reported associations between brain iron deposits and cognitive status, and cardiovascular and neurodegenerative diseases in older individuals, but the mechanisms underlying these associations remain unclear. We explored the associations between regional brain iron deposits and different factors of cognitive ability (fluid intelligence, speed and memory) in a large sample (*n* = 662) of individuals with a mean age of 73 years. Brain iron deposits in the corpus striatum were extracted automatically. Iron deposits in other parts of the brain (i.e., white matter, thalamus, brainstem and cortex), brain tissue volume and white matter hyperintensities (WMH) were assessed separately and semi-automatically. Overall, 72.8 % of the sample had iron deposits. The total volume of iron deposits had a small but significant negative association with all three cognitive ability factors in later life (mean *r* = −0.165), but no relation to intelligence in childhood (*r* = 0.043, *p* = 0.282). Regression models showed that these iron deposit associations were still present after control for a variety of vascular health factors, and were separable from the association of WMH with cognitive ability. Iron deposits were also associated with cognition across the lifespan, indicating that they are relevant for cognitive ability only at older ages. Iron deposits might be an indicator of small vessel disease that affects the neuronal networks underlying higher cognitive functioning.

**Electronic supplementary material:**

The online version of this article (doi:10.1007/s11357-015-9837-2) contains supplementary material, which is available to authorized users.

## Introduction

As people grow older, iron accumulates, mainly in the form of hemosiderin, in several brain regions and cell types (Valdés Hernández et al. 2012; Ward et al. 2014). The causes of these iron deposits, and their consequences for human cognitive ageing, remain unclear. Initial evidence indicates that they are related to lower cognitive ability in later life (Penke et al. 2012) and to a variety of neurological diseases (e.g., Brass et al. 2006). In the present study, we analyse data from a large sample of individuals aged around 73 years, building a picture of where in the brain most iron deposits are found, and testing their relations with multiple cognitive abilities and vascular risk factors.

Several sources of iron deposition have been described in older and cognitively normal brains. Macroscopic mineralised ‘pools’, predominant in ferritin and hemosiderin, mainly localised in and around the small lenticulostriate arterioles of the corpus striatum (Casanova and Araque 2003; Aquino et al. 2009), are the main contributors to the abnormal iron accumulation detectable using magnetic resonance images (MRI). This deposition gradually occurs and has been mainly attributed to dysfunctional brain iron regulatory mechanisms including abnormal permeability of the vessel walls and glial cell dysfunction (McCarthy and Kosman 2014). Small chronic haemorrhages, in different forms and locations, are also sources of macroscopic iron deposition: brain microbleeds and superficial siderosis are two of the types most commonly found in older brains. Brain microbleeds, tiny deposits of blood degradation products contained within macrophages, are in close spatial relationship with structurally abnormal vessels (Martinez-Ramirez et al. 2014). Superficial siderosis manifests in the subpial layers of the brain as hemosiderin accumulation, due to recurrent and persistent bleeding into the subarachnoid space (Kumar 2009).

Previous studies have found associations between higher levels of brain iron deposits in deep grey matter structures and lower cognitive ability (Sullivan et al. 2009; van Es et al. 2008; Daugherty et al. 2015; Rodriguez et al. 2013), as well as neurodegenerative diseases (Thompson et al. 2001; Wallis et al. 2008; McNeill et al. 2008; Kruer et al. 2012; Brass et al. 2006). Microbleeds have been associated with cerebrovascular diseases (Fazekas et al. 1999; Cordonnier et al. 2007). A previous study of a subset of the cohort analysed in this paper (the Lothian Birth Cohort 1936 (LBC1936); Penke et al. 2012) showed an association between the volume of iron deposits (primarily in the basal ganglia) and poorer general cognitive ability. However, with data from only 143 individuals, it had relatively low statistical power, increasing the chances of erroneous results. In addition, it did not analyse other variables, such as vascular risk factors, and white matter hyperintensities (WMH) that could be potentially related to brain iron deposition. In this paper, we report data on the iron deposition levels and locations for the full cohort (*n* = 676 with relevant MRI data), along with the relations of the volume of iron deposits to cognitive and vascular health.

In addition, the Lothian Birth Cohort 1936 has available a measure of childhood intelligence, taken at age 11. In the subsample analysed by Penke et al. (2012), cognitive ability from early life predicted the volume of iron deposition in the brain at age 73. The latter was related to lifetime cognitive ability (that is, iron deposit volume related to later-life cognitive ability after controlling for early life cognitive ability). Whether these associations hold for a bigger sample needs to be investigated.

Here, we also investigate a second hypothesis, related to WMH: hyperintensities observed in the white matter and subcortical grey matter on fluid attenuation inversion recovery (FLAIR) and T2W structural MRI; which are common in older brains and may have similar associations with indicators of cognitive ability as brain iron deposits (Valdés Hernández et al. 2013a; Wardlaw et al. 2013). Iron deposits in the globus pallidus have been associated with the total brain WMH volume (Yan et al. 2013). A detailed characterisation of corpus striatum (i.e., basal ganglia and internal capsule) multifocal T2*W hypointensities on MRI from an ageing sample concluded that their spatial distribution and multifocal morphology suggest they could be associated with proximal mineralised lenticulostriate arteriolar walls and perivascular structures (Glatz et al. 2013). Iron deposits may, therefore, have a mainly vascular origin, like the WMH found in deep grey and white matter (Kim et al. 2008). However, it is unclear whether the presence of iron deposits has effects on cognitive ability beyond those of WMH. In this study, we test whether iron deposits are still related to cognitive ability even after taking into account the association with WMH.

After describing the regional locations of the brain iron deposits in our sample, we focus on three questions. First, do iron deposits predict cognitive ability, and do they do so even after control for a range of health factors? We test this hypothesis using a series of linear regression models. Second, do iron deposits still relate to cognitive ability after taking into account the association with WMH? If separate effects are found, this would be evidence consistent with additive detrimental effects of iron deposits and WMH on cognitive abilities. If the association with iron deposits is no longer present after adjusting for WMH, it would imply that iron deposits are epiphenomena of other processes and are not themselves predictive of cognitive ability. Third, we estimate models that test whether iron deposits relate to lifetime cognitive ability, using the age-11 intelligence data in our sample as a control variable. If iron deposits relate to cognition across the life course, we would expect still to find an association between iron deposit volume and cognitive ability at old age when we account for childhood intelligence like we found before on a smaller sample.

## Materials and methods

### Subjects

From the Lothian Birth Cohort 1936 (LBC1936), which comprises community-dwelling surviving members of the Scottish Mental Survey of 1947 (Deary et al. 2007), 700 participants (328 females and 372 males) had an MRI brain scan at mean age 72.7 years old (SD 0.7, range 71.1 to 74.3). From the 700 brain image sets, 676 had the relevant sequences to assess brain iron deposition and WMH. Three scans were additionally excluded due to the presence of extensive hemosiderin deposition caused by previous haemorrhages. However, the final sample size slightly varied across analyses depending on the cognitive and imaging data available. Each participant’s history of diabetes, hypertension, hypercholesterolaemia, cardiovascular disease and stroke was taken from their self-reported medical history. Written informed consent was obtained from all participants under protocols approved by the Lothian (REC 07/MRE00/58) and Scottish Multicentre (MREC/01/0/56) Research Ethics Committees.

### MRI scans

MRI scans were acquired using a 1.5 T GE Signa Horizon HDxt clinical scanner (General Electric, Milwaukee, WI, USA) operating in research mode and using a self-shielding gradient set with maximum gradient of 33 mT/m and an 8-channel phased-array head coil. The imaging protocol is fully described in (Wardlaw et al. 2011). For this particular study, we used the coronal T1W volumes acquired with a 3D inversion recovery prepared fast gradient echo sequence (TR/TE/TI = 9.7:3.984:500 ms, flip angle *α* = 8 °, bandwidth 15.63 kHz, voxel size 1 × 1 × 1.3 mm^3^), the axial FLAIR volumes (TR/TE/TI = 9000:140:2200 ms, bandwith 15.63 kHz, voxel size 1 × 1 × 4 mm^3^) and the axial T2*W volumes acquired with a 2D gradient echo sequence (GRASS, TE/TR = 15:940 ms, flip angle *α* = 20 °, bandwidth 12.5 kHz, voxel size 1 × 1 × 2 mm^3^). None of these sequences had interslice gap and, for all, the FOV in the acquisition plane was 256 × 256 mm^2^.

### Brain iron deposits

For extracting and quantifying the brain volume occupied by iron deposits, we used the pre-processing pipeline described in Glatz et al. (2012, 2015) and applied computational methods that use T1W and T2*W MRI sequences to ensure that we only include non-calcified regions rich in methemoglobin, hemosiderin and/or ferritin (Valdés Hernández et al. 2012, 2014; Vymazal et al. 1999).

#### Diffuse and morphometrically heterogeneous iron deposits in the corpus striatum

Multifocal T2*W hypointensities in the structures of the corpus striatum that can appear uniformly spatially distributed on a morphometrically irregular cluster or group of clusters on structural MRI, were assessed fully automatically using the method described in Glatz et al. (2015), freely available at https://github.com/aglatz/mineral-deposit-segmentation-pipeline/tree/master/libBRIC/mineral-deposit-segmentation, and reported Jaccard similarity index = 0.62 ± 0.40 (Glatz et al. 2015). Briefly, T2*W hypointensities are segmented with thresholds derived with an adaptive outlier detection method from the bivariate T2*W/T1W intensity distributions in each structure of the pre-processed T1W/T2*W images. Artefacts are reduced by filtering connected components in the binary masks of the T2* hypointense clusters based on their standardised T2*W intensity variance and appearance on T1W MRI. The results of the automatic segmentation were individually checked by an experienced analyst blinded to any other imaging, cognitive and neurological information and manually rectified in the few cases it was required.

#### Iron deposits in the brainstem, white matter, thalamus and cortex

T2*W hypointensities in the brainstem, white matter, thalamus and cortex were separately identified, extracted and quantified on the pre-processed T2*W scans semi-automatically using the ‘Object Counter’ module in Analyze^TM^ 10.0 following the process described in Valdés Hernández et al. (2011, 2013a). A slice was selected where the T2*W hypointensities appear, ideally with a variety of shapes and intensities to adjust the intensity threshold, starting from zero to less than half of the median intensity value of the normal-appearing white matter. An estimated maximum and minimum size of the hypointense ‘objects’ was then adjusted interactively. Binary masks of regional iron deposits were obtained out from the intersection between the binary masks obtained from this semi-automatic process and the regional regions-of-interest masks obtained from the pre-processing pipeline. Manual editing followed the protocol described and validated in (Penke et al. 2012; Valdés Hernández et al. 2011; Glatz et al. 2013).

#### Brain microbleeds and macrohaemorrhages

Brain microbleeds were visually assessed by an experienced neuroradiologist using the Brain Observer Micro Bleed Scale (BOMBS) (Cordonnier et al. 2007). Macrohaemorrhages were also noted using a structured template (Wardlaw et al. 2011). The sphericity and size of the iron deposits computationally assessed was used to evaluate the contribution of the ‘possible’ brain microbleeds on the overall ID load, and, therefore on the associations evaluated. The one-to-one correspondence between visually detected ‘certain’ microbleeds and the small spherical T2*W hypointensities computationally extracted was reported and examined. The presence of iron deposits in these regions with other possible aetiology (e.g., superficial siderosis, previous haemorrhage) was annotated.

### Intracranial, brain tissue and WMH volume measurements

Volumes of the intracranial space (ICV), brain tissue and WMH segmented as described in Wardlaw et al. (2011), were used in the analyses. Briefly, the ICV (i.e., contents within the inner skull table including brain tissue, cerebrospinal fluid, veins and dura), with an inferior limit on the axial slice just superior to the tip of the odontoid peg at the foramen magnum, was extracted semi-automatically using the T2*W sequence, with the Object Extraction Tool in Analyze^TM^ 10.0 followed by manually editing. WMH were segmented semi-automatically on the quantised colour image obtained after fusing co-registered FLAIR and T2*W sequences, mapping them in green and red respectively, and applying minimum variance quantisation. This technique is described and validated elsewhere (Valdés Hernández et al. 2010) and implemented by MCMxxxVI_ALE: tool freely available from (www.sourceforge.net/projects/bric1936). On a randomly selected subsample of 20 individuals from this cohort, it had a substantial agreement (Jaccard similarity index = 0.61, 95 % confidence interval (CI) = 0.42) with WMH reference segmentations. ICV was used to correct WMH volumes for head size. All these measurements were also performed by a trained image analyst blinded to participants’ clinical and demographic information.

### Cognitive testing and cognitive variables

We used cognitive measures obtained at the same time as the MRI scan (mean age 72.7, SD 0.7 years). These cognitive variables were fluid intelligence (*g*-fluid), general processing speed (*g*-speed) and general memory (*g*-memory). These latent general cognitive ability measures were generated using principal component analysis from batteries of well-validated cognitive tests. For *g*-fluid, we used six subtests of the WAIS-III^UK^ (Wechsler 1998): Digit Symbol Substitution, Digit Span Backward, Symbol Search, Letter-Number Sequencing, Block Design & Matrix Reasoning. *g*-memory was derived from five subtests from the WMS-III^UK^ (Wechsler 1998)—Logical Memory Total Immediate & Delayed Recall, Verbal Paired Associates Immediate & Delayed Recall, and Spatial Span Total Score—along with two subtests from the WAIS-III^UK^: Letter-Number Sequencing and Digit Span Backward. *g*-speed was obtained from two reaction time tests—Simple Reaction Time and Choice Reaction Time—an Inspection Time test, and two WAIS-III^UK^ subtests: Digit Symbol Substitution and Symbol Search.

### Statistical analyses

For all reported analyses, the total and regional iron and WMH volumes were standardised by ICV and expressed in percentage with respect to it. Age in days at the time of the MRI scan and/or cognitive test was entered as a covariate in all models to control for individual differences in chronological age that may confound the analysis of individual differences in brain measurements and/or of brain-cognition associations. The volumes of total iron deposits and white matter hyperintensities were positively skewed; we thus log-transformed these variables before including them in the analysis. Analyses were carried out within R. Missing value analysis revealed patterns of missing data were random.

## Results

### Sample characteristics

The descriptive statistics of the imaging and cognitive variables involved in the analyses are given in Table 1. Approximately half (48.6 %) of the participants from the sample had hypertension, 10.8 % had diabetes, 41.2 % had hypercholesterolaemia, 27.2 % had history of cardiovascular disease and 17.7 % had history of stroke. Details of the measured values of the continuous cardiovascular biomarkers on this sample have been reported previously (Wardlaw et al. 2014). Iron deposits in the corpus striatum, with median volume of 0.03 ml, were found in 477 participants (70.8 % of the sample). Iron deposits in the brainstem were found in 87 participants (12.9 %), in the cortex in 10 (1.5 %), in the white matter in 42 (6.2 %) and in the thalamus in 8 (1.2 %). Iron deposits were present in 72.8 % of the sample (490 participants; Table S1 Supplementary Material).

**Table 1:** Descriptive statistics for the cognitive tests and brain volumetric variables

Variable type	Parameter	Number	Mean (SD)
Cognitive test	Digit symbol substitution	656	56.34 (12.20)
	Digit span backward	658	7.84 (2.31)
	Symbol search	656	24.69 (6.14)
	Letter-number sequencing	658	10.97 (3.03)
	Block design	656	34.16 (10.08)
	Matrix reasoning	656	13.45 (4.90)
	Logical memory (total)	658	37.23 (9.07)
	Verbal paired associates (total)	642	4.58 (1.96)
	Spatial span	655	7.35 (1.37)
	Simple reaction time (ms)	657	0.27 (0.05)
	Choice reaction time (ms)	657	0.64 (0.09)
	Inspection time	645	111.36 (11.77)
Brain volumetric	Total brain vol. (ml)	676	1123.98 (106.86)
	Intracranial vol. (ml)	679	1450.98 (140.57)
	Total iron deposit vol. (ml)	651	0.04 (0.20)
	Total WMH vol. (ml)	655	7.70 (13.35)

Sample restricted to individuals with usable brain imaging data on hyperintensities

*WMH* white matter hyperintensities

The median total volume of iron deposits was 0.04 ml. The maximum volume of iron deposits found in the corpus striatum was 2.8 ml, in the brainstem 0.75 ml, in the cortex 0.44 ml, in the white matter 1.4 ml and in the thalamus 0.12 ml. These figures were affected by previous macrohaemorrhages in the basal ganglia, cortex and white matter. However, only seven participants had iron deposits due to previous macrohaemorrhages in these regions.

#### Characterisation of iron deposits in the sample

**Fig. 1:**
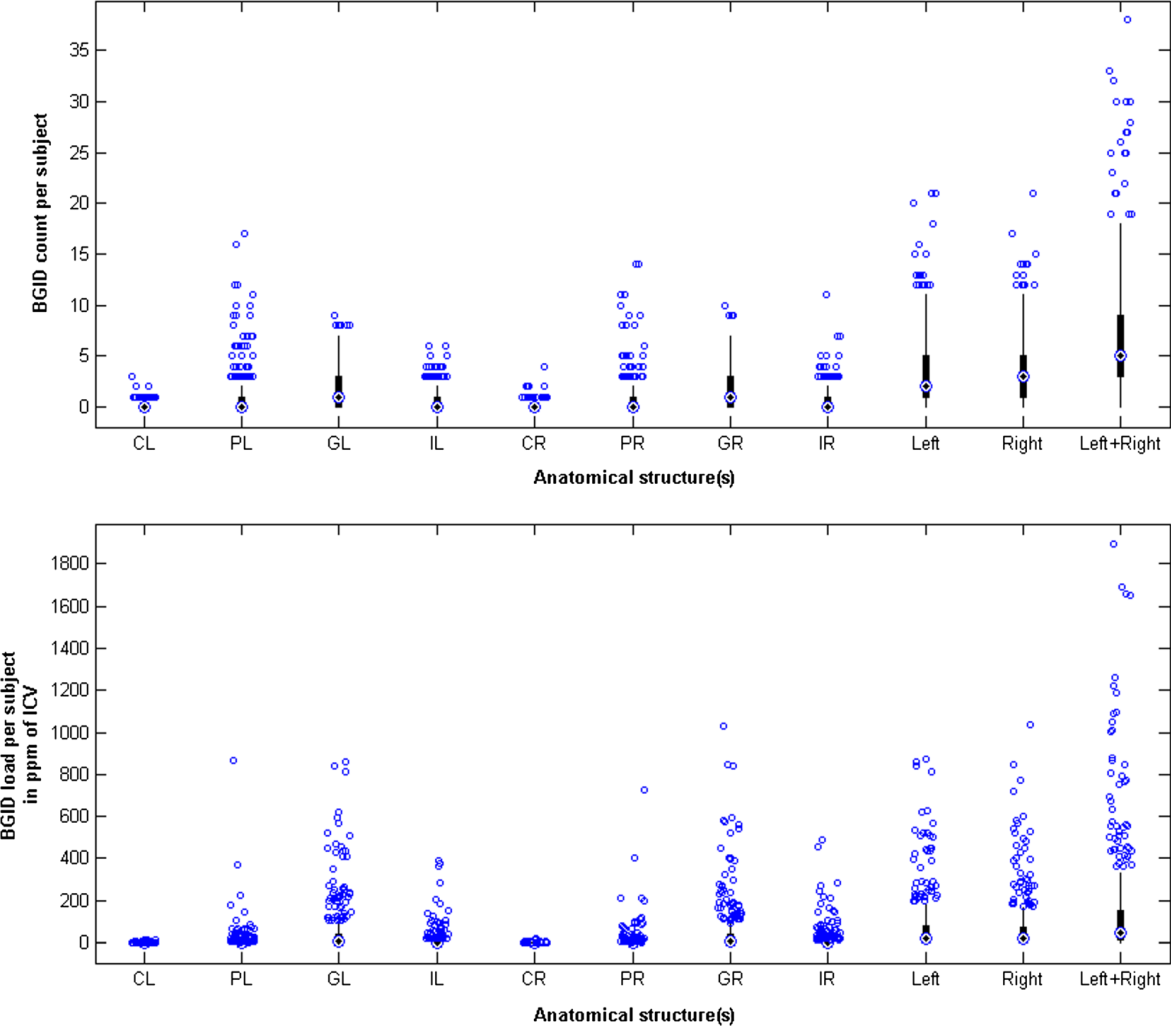
Number of individual clusters and load of iron deposits (expressed in ppm of ICV) in the structures of the corpus striatum for 421/474 datasets. From *left to right in the x-axis*: left caudate nucleus (*CL*), left putamen (*PL*), left globus pallidus (*GL*), left internal capsule (*IL*), right caudate nucleus (*CR*), right putamen (*PR*), right globus pallidus (*GR*), right internal capsule (*IR*), total volume and count on the left hemisphere (*left*), on the right (*right*) and in both hemispheres (*left* + *right*)

**Fig. 2:**
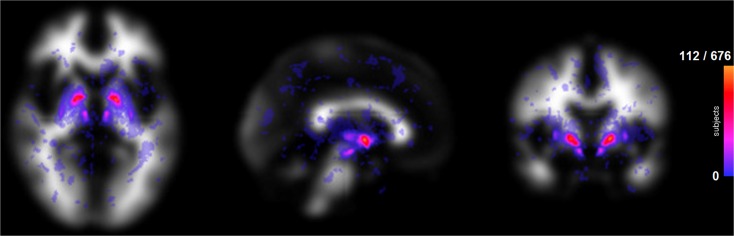
Distributional map of iron deposits on the sample. Maximum intensity projection of the iron deposits on (from *left to right*) mid-axial, sagittal and coronal views

Of the 10 participants who had iron deposits in the cortex, only 2 presented radiological evidence of a mild superficial siderosis defined as per Kumar (2009). The rest were small spherical T2*W hypointensities identified as microbleeds. Twelve scans presented microbleeds on the border-zone between white matter and cortex. The volume and number of ID clusters in the globus pallidus were more prominent than in other regions of the corpus striatum (Fig. 1). The corpus striatum iron deposition load was balanced in both hemispheres (Figs. 1 and 2). The anatomical distribution pattern of iron deposits was consistent with those reported by histopathological studies (Ogg and Steen 1998; Morris et al. 1992).

#### Presence of vascular risk factors, history of cardiovascular disease, previous stroke and gender differences in relation to regional iron deposition load

Supplementary Table S2 shows the results of testing the null hypotheses that the distribution of regional iron deposits was the same for men and women and across subjects with presence or not of vascular risk factors, history of cardiovascular disease or previous stroke. The volume of iron deposits in the corpus striatum was significantly higher for participants with hypercholesterolaemia (*p* = 0.002) and history of stroke (*p* = 0.006) vs. those without. Iron deposits in the brainstem were significantly higher for men than for women (mean volume 0.020 ml for men vs. 0.010 ml for women; *p* = 0.002), and for participants with than without diabetes (*p* = 0.016), cardiovascular disease (*p* = 0.036) and history of stroke (*p* = 0.012). Despite few participants having iron deposits in the white matter, cortex and thalamus, iron deposits in these regions (i.e., considering each region separately) were significantly related with history of stroke (*p* < 0.020). Combining together the iron deposits in white matter, cortex and thalamus, the difference between participants with vs. without hypercholesterolaemia was also significant (*p* = 0.029). All participants with a history of stroke had iron deposits, and all participants with iron deposits in the white matter had at least one stroke. Neither regional nor total volumes of iron deposition on participants with hypertension significantly differed from those on participants reported as normotensive or with low blood pressure levels. Results for the total volume of iron deposits, however, were mainly influenced by the prevalence of iron deposits in the brainstem and corpus striatum. The median total ID volume of participants with hypercholesterolaemia was twice as high as of those with normal cholesterol (0.056 ml, inter-quartile range (IQR) = 0.219 ml vs. 0.028 ml, IQR = 0.164 ml, respectively). Similar proportion in the total ID volume was observed between participants with diabetes vs. those without it, with median total ID volumes of 0.067 ml, IQR = 0.285 ml vs. 0.038 ml, IQR = 0.184 ml, respectively.

### Bivariate relations

A correlation matrix showing the bivariate relations among the cognitive ability measures, brain variables and vascular risk factors is shown in Table 2. As expected, the cognitive measures were highly intercorrelated. All three later-life cognitive factors were significantly negatively correlated with total iron deposit volume (average *r* = −0.165) and total WMH volume (average *r* = −0.126). However, there was no significant correlation between age 11 cognitive ability and either iron deposits or WMH. Volumes of both iron deposits and WMH were higher in individuals with hypercholesterolaemia and stroke, and WMH volume was higher in the presence of hypertension. There were no relations between the brain measures and either diabetes or cardiovascular disease. There was no significant correlation between the total volume of iron deposits and the total volume of WMH. Finally, we found that there was no difference in total iron deposit volume between male and female cohort members (*t*(648.35) = 1.37, *p* = 0.17); the same was found for total WMH volume (*t*(603.08) = 0.15, *p* = 0.88).

**Table 2:** Correlation matrix for the primary variables used in the analysis

Variable	(1)	(2)	(3)	(4)	(5)	(6)	(7)	(8)	(9)	(10)
(1) Age 11 MHT	–									
(2) Fluid intelligence	0.574***	–								
(3) Speed	0.402***	0.760***	–							
(4) Memory	0.528***	0.689***	0.490***	–						
(5) Total iron deposit volume	−0.043	−0.177***	−0.172***	−0.145***	–					
(6) White matter hyperintensity volume	−0.075	−0.156***	−0.144***	−0.079*	0.037	–				
(7) Hypertension^†^	−0.019	−0.095*	−0.087*	−0.003	0.059	0.122**	–			
(8) Diabetes^†^	−0.081	−0.104**	−0.126**	−0.089*	0.072	0.035	0.141***	–		
(9) Hypercholesterolaemia^†^	−0.023	−0.057	−0.089*	−0.035	0.101**	0.082*	0.286***	0.176***	–	
(10) Cardiovascular disease^†^	−0.065	−0.018	−0.017	−0.002	0.014	0.019	0.174***	0.123***	0.206***	–
(11) Stroke^†^	−0.058	−0.085*	−0.075	−0.061	0.150***	0.171***	0.139***	0.088*	0.142***	0.060

Statistically significant correlations in italics. All brain and cognitive variables controlled for age and sex before calculation of correlations. Correlations between continuous variables calculated using Pearson correlations; correlations between continuous and categorical variables calculated using point-biserial correlations; correlations between categorical variables calculated using the phi coefficient

*MHT* moray house test; all variables aside from age 11 MHT measured at age ∼73

*p < 0.05; **p < 0.01; ***p < 0.001

^†^ = dichotomous variable; all other variables continuous

### Multivariate regression models

We first tested the hypothesis that iron deposits would be related to cognitive ability even after taking into account vascular health. The results of the three regression models predicting total iron deposit volume (one including each of the three cognitive ability factors: *g*-fluid, *g*-speed and *g*-memory) are shown in Table S3 in the Supplementary Material. In summary, in all three models, cognitive ability was significantly related to iron deposit volume even after control for all health factors (mean *β* = −0.119). After multivariate control, the only health factor that was consistently related to iron deposit volume was the participant’s previous history of stroke; that is, the significant bivariate correlation shown in Table 1 remained significant after inclusion in the model of the other vascular risk factors.

In the second set of regression models, we used cognitive ability as the dependent variable and included both iron deposits and WMH as predictors, alongside the vascular health variables. These results are shown in Table 3. For all three cognitive factors, both iron deposits and WMH had significant associations with cognitive ability: that is, they had separable contributions to explaining variance in *g*-fluid, *g*-speed and *g*-memory, although the effect sizes were modest for both iron deposits (mean *β* = −0.140) and WMH (mean *β* = −0.138).

**Table 3:** Linear regression models predicting levels of fluid intelligence (model 1), speed (model 2) and memory (model 3) including iron deposit and white matter hyperintensity volumes. Italic coefficients were statistically significant at *p* < 0.05

Predictor	Model 1				Model 2				Model 3			
	*g*-fluid				*g*-speed				*g*-memory			
	(*n* = 644)				(*n* = 636)				(*n* = 632)			
	*β*	SE	*p*	Adj. *R* ^2^	*β*	SE	*p*	Adj. *R* ^2^	*β*	SE	*p*	Adj. *R* ^2^
Age	−*0.120*	*0.038*	*0.002*		−*0.140*	*0.038*	<*0.001*		−*0.083*	*0.040*	*0.035*	
Sex	0.020	0.038	0.595		0.054	0.040	0.151		0.092	0.039	0.020	
Total iron deposit volume	−*0.124*	*0.038*	*0.001*		−*0.190*	*0.038*	*0.013*		−*0.106*	*0.039*	*0.007*	
Total WMH volume	−*0.138*	*0.039*	<*0.001*		−*0.190*	*0.039*	<*0.001*		−*0.086*	*0.040*	*0.034*	
Hypertension	−0.098	0.080	0.222		−0.052	0.080	0.507		0.073	0.083	0.377	
Diabetes	−*0.279*	*0.126*	*0.027*		−*0.323*	*0.125*	*0.010*		−*0.272*	*0.132*	*0.039*	
Hypercholesterolaemia	0.013	0.081	0.869		−0.075	0.081	0.358		−0.011	0.085	0.894	
Cardiovascular disease	−0.002	0.087	0.977		0.008	0.087	0.930		−0.001	0.091	0.992	
Stroke	−0.060	0.102	0.556		0.001	0.102	0.994		−0.074	0.106	0.483	
				0.065				0.089				0.036

*WMH* white matter hyperintensity

Finally, we tested whether iron deposits were related to lifetime cognition, by including in the model the measure of age 11 cognitive ability (Table 4). We found that the total volume of iron deposits still predicted all three cognitive variables after control for age 11 cognitive ability (mean *β* = −0.101), and WMH still predicted *g*-fluid and *g*-speed (mean *β* = −0.118), but no longer significantly predicted *g*-memory (*β* = −0.047, *p* = 0.193). Combined with the lack of correlation between age 11 intelligence and iron deposits noted above, this indicated that iron deposits were relevant to later-life cognitive ability, and were not simply related to pre-existing cognitive ability from childhood.

**Table 4:** Linear regression models predicting levels of fluid intelligence (model 1), speed (model 2) and memory (model 3), controlling for childhood cognitive ability (and thus assessing lifetime cognition). Italic coefficients were statistically significant at *p* < 0.05

Predictor	Model 1				Model 2				Model 3			
	*g*-fluid (*)				*g*-speed (*)				*g*-memory (*)			
	(*n* = 644)				(*n* = 636)				(*n* = 632)			
	*β*	SE	*p*	Adj. *R* ^2^	*β*	SE	*p*	Adj. *R* ^2^	*β*	SE	*p*	Adj. *R* ^2^
Age	−*0.087*	*0.032*	*0.007*		−*0.130*	*0.036*	<*0.001*		−0.054	0.034	0.115	
Sex	−0.048	0.032	0.141		0.008	0.036	0.817		0.023	0.035	0.509	
Age 11 MHT	*0.550*	*0.032*	<*0.001*		*0.364*	*0.036*	<*0.001*		*0.522*	*0.034*	<*0.001*	
Total iron deposit volume	−*0.114*	*0.032*	<*0.001*		−*0.097*	*0.036*	*0.007*		−*0.091*	*0.034*	*0.008*	
Total WMH volume	−*0.088*	*0.033*	*0.007*		−*0.147*	*0.036*	<*0.001*		−0.046	0.034	0.193	
Hypertension	−0.120	0.067	0.075		−0.082	0.075	0.277		0.054	0.072	0.449	
Diabetes	−0.168	0.106	0.112		−*0.261*	*0.119*	*0.028*		−0.163	0.114	0.154	
Hypercholesterolaemia	0.027	0.069	0.693		−0.057	0.077	0.461		−0.010	0.074	0.892	
Cardiovascular disease	0.029	0.074	0.697		0.007	0.083	0.930		0.044	0.079	0.583	
Stroke	−0.038	0.086	0.654		0.045	0.097	0.639		0.038	0.091	0.681	
				0.368				0.220				0.300

*MHT* moray house test score, *WMH* white matter hyperintensity volume

*Refers to change from age 11 to age 72.7

## Discussion

### Associations between brain iron deposits, cognitive ability, WMH and vascular health

Our results, from analyses of a large sample of older individuals show a significant and negative association between the total volume of iron deposits in the brain and a variety of cognitive measures. These associations were above and beyond those of WMH, were still present after controlling for a number of vascular health factors, and were unrelated to pre-existing cognitive ability.

The findings build upon the results of some previous exploratory studies. For instance, Sullivan et al. (2009) used quantitative estimates of regional iron concentration from 10 healthy elderly subjects and found poorer cognitive ability in those with higher levels of iron deposition. Penke et al. (2012) examined mineral depositions with iron content from a subsample of 143 participants from the cohort analysed in the present study. In that subsample, there was a relation between childhood intelligence and iron deposition. The larger analysis in the present study shows that this is not, in fact, the case: there was only a small, non-significant association between iron deposit volume and early life cognitive ability in the full LBC1936 sample (*r* = −0.043, *p* = 0.282).

At the other end of the life course, however, we found consistent relations between iron deposit volume and cognitive ability. There were associations, of broadly similar effect size, between total iron deposit volume and factors of fluid intelligence, processing speed and memory, all indexed by a wide range of high-quality cognitive tests. Including the total volume of WMH as a predictor in the model as well as iron deposit volume hardly affected the results: these two types of age-related brain abnormality had unique, separable associations with cognitive ability. Controlling for age 11 cognitive ability reduced the effect sizes somewhat, but the association of iron deposit volume with cognitive ability remained significant in every case. This confirmed that the association of iron deposits with cognitive ability is a later-life phenomenon, and is not dependent on early life cognitive levels.

In this birth cohort, iron deposits were more common in participants with previous evidence (episodic or imaging) of stroke; indeed, this relation remained significant after multivariate statistical control for four other related health concerns (hypertension, diabetes, hypercholesterolaemia and cardiovascular disease). Hypercholesterolaemia was, in order of effect size, the second most influential factor on total brain iron accumulation after history of stroke. Participants with hypercholesterolaemia had significantly more iron deposits in the corpus striatum than participants with normal levels of cholesterol, a finding not observed in relation with iron deposits of any other brain region. All this suggests that iron deposits of the corpus striatum might be a better metabolic biomarker than iron deposits in other brain regions.

In all models, the associations between iron deposits (and WMH) and cognitive ability remained significant after controlling for the presence or absence of five health risk conditions that relate to vascular health (hypertension, diabetes, hypercholesterolaemia, cardiovascular disease and occurrence of a previous stroke). It may be that these diseases (in particular diabetes; see Table 3) exert separate effects on cognitive ability from any effects of iron deposition; it may also be that both diabetes and iron deposits are indicators of a more distal factor that itself lowers cognitive ability. Future research should aim to test whether there are health and lifestyle factors (such as smoking) that significantly attenuate the association of iron deposit volume with cognitive ability in models similar to ours; that is, they should test for factors that may cause additional iron to be produced in the brain.

### Strengths and limitations

We used visual ratings of microbleeds (Cordonnier et al. 2007), a validated quantitative approach to assess regional multifocal T2*W hypointensities as a surrogate of the multifocal brain ID load (Glatz et al. 2013, 2015; Valdés Hernández et al. 2011, 2014), a validated semi-automatic WMH segmentation method (Valdés Hernández et al. 2010, 2013b) and measures obtained from comprehensive batteries of cognitive tests, in an attempt to address our main research questions. In addition to the robust assessment of the imaging markers, other strengths of this paper are the analysis of the role that WMH play in the effect that iron deposits have in cognition, explored here for the first time, and the use of several validated measures of cognitive ability in a large cohort of community-dwelling older individuals to explore the association between intelligence and iron deposits in later life.

The sample participants were of similar age and relatively healthy; thus, the anatomical distribution of the iron load in this cohort does not allow us to explore the influence of age in the iron accumulation process. However, it provides representative evidence of the ID distribution and its correlates in a healthy septuagenarian European sample, relevant for epidemiological and ageing studies. In this cohort, iron deposits in the white matter, thalamus and cortex were found only on few individuals. This meant that we restricted our analyses to the total iron deposit volume and did not examine any potential specific links between iron deposits in particular regions and cognitive ability. Replication of this analysis on large studies of patients with conditions like stroke or dementia is necessary.

The fact that the true volume of iron accumulation in tissues cannot accurately be determined using MRI techniques is a caveat of the present study. The volumetric measurements of iron are estimates obtained from measuring the MRI signal produced by the effect of iron accumulation in tissues. Studies show that, contrary to the strong linear correlation between iron concentration and the effective transverse relaxation 1/T2* in the grey matter, in white matter, this correlation is much weaker (Langkammer et al. 2012; Schweser et al. 2011), affecting the accuracy of computational measurements. This has been attributed to different factors: different levels of myelin (Langkammer et al. 2012), the relative volume and oxygenation state of blood (Lee et al. 2010), chemical exchange between water and macromolecular protons (Luo et al. 2010; Zhong et al. 2008), the orientation of underlying white matter fibres with respect to the main magnetic field (Denk et al. 2011) and variations in the macromolecular mass fraction (Mitsumori et al. 2009). To partially overcome this limitation, we report neuroradiological visual count of microbleeds: a form of iron deposition predominantly found on the white matter, which is considered the gold standard in the study of iron accumulation-related pathologies, along with validated automatic and semi-automatic assessments, all visually checked and manually rectified when deemed necessary.

Moreover, old micro-/macro-haemorrhages can appear as calcifications on conventional MRI (Bradley 1993; Valdés Hernández et al. 2014) and, therefore, were not included in the volume of brain iron deposition. Although there is always an overlap in the intensities that indicate the presence of calcified and non-calcified iron deposits due to the coalescence of both minerals in many regions as a consequence of metabolic processes and the degradation of the non-soluble iron molecules (Haacke et al. 2005), the sequence combination of T1W and T2*W (Valdés Hernández et al. 2014) and the filter implemented as part of the automatic method that assessed the corpus striatum iron deposits (Glatz et al. 2015), ensured that heavily calcified regions were not included. This is a difference with the computational method used in Penke et al. (2012). Although it is known that these calcified regions are strongly and directly associated with the iron deposits assessed (Valdés Hernández et al. 2014), it has been proposed that calcification is the latest stage in the iron degradation and accumulation processes with a neuroprotective effect (Casanova and Araque 2003). We would not predict that the inclusion of these old micro-/macro-haemorrhages would have changed the associations found between iron deposition and cognitive abilities at old age, but it may be possible that the role of early life general cognitive ability in the vascular degeneration that occurs at old age, represented by the presence of brain iron deposits, may have been underestimated.

Given the time elapsed between the participants’ assessments (i.e., along 3 years), along which the MR scanner was serviced twice and upgraded once, the average intensity value from the T2*W images which could express relative tissue/regional differences in iron content, was not measured. Our scanning protocol did not produce quantitative R2* (i.e., 1/T2*) values to determine the regional iron concentration. Without these additional measures it is difficult to estimate the degree in which low-level disperse oxidative stress, produced on larger volumes of low iron concentration, affects cognition as opposed to the effect that a focal high-concentration source has, which is reported in this manuscript.

### Implications for further research

Although the total iron measures we obtained may be dominantly influenced by biologically inert forms as part of ferritin, hemosiderin and other macromolecules, they might be predictive for bioactive iron compounds and, as such, have relevance for the study of ageing and neurodegenerative processes (Pujol et al. 1992; Ward et al. 2014). In our normal ageing cohort, these iron deposits tended to be found in the same brain regions in which iron accumulates in neurodegenerative diseases like Alzheimer’s and Parkinson’s (Collingwood et al. 2008; Vymazal et al. 1999). This constitutes further evidence that ageing and some neurodegenerative pathologies share similar brain mechanisms involving iron. If brain iron patterns are characteristic of disease or disease stages, this might have implications for prediction and diagnosis.

## Electronic supplementary material

Below is the link to the electronic supplementary material.

ESM 1 (DOCX 21 kb)
