# Supplementary material for: Brain iron deposits and lifespan cognitive ability
Source: Age (Dordr). 2015 Sep 17;37(5):100. doi: 10.1007/s11357-015-9837-2 (PMC5005839; doi:10.1007/s11357-015-9837-2)
Supplement: Supplementary file 1 — (DOCX 21 kb) [file 11357_2015_9837_MOESM1_ESM.docx]

**Brain iron deposits and cognitive change across the lifespan**

**Supplementary material**

*Table S1*. Detailed descriptive statistics of the imaging markers split by gender

| **Parameter** | **Median** | | | **Interquartile range / Range** | | |
| --- | --- | --- | --- | --- | --- | --- |
|  | **Men** | **Women** | **Total** | **Men** | **Women** | **Total / Range** |
| Corpus striatum iron deposits volume (ml) | 0.039 | 0.032 | 0.035 | 0.159 | 0.161 | 0.159 / 2.768 (0 – 2.768) |
| Total ID volume (ml) | 0.046 | 0.036 | 0.040 | 0.201 | 0.187 | 0.196 / 3.224 (0 – 3.224) |
| WMH volume (ml) | 7.870 | 7.402 | 7.704 | 13.218 | 13.866 | 13.348 / 98.378 (0 – 98.378) |
|  | **Incidence (i.e. No. participants with iron deposits on the region)** | | | **Volume range (ml)** | | |
| Corpus striatum iron deposits | 260 / 358 | 217 / 316 | 477 / 674 | 2.768 | 2.670 | 2.768 (0 – 2.768) |
| BS iron deposits | 61 / 358 | 26 / 316 | 87 / 674 | 0.748 | 0.554 | 0.748 (0 – 0.748) |
| Cortex iron deposits | 9 / 358 | 1 / 316 | 10 / 674 | 0.440 | 0.004 | 0.440 (0 – 0.440) |
| WM iron deposits | 23 / 358 | 19 / 316 | 42 / 674 | 1.404 | 0.946 | 1.404 (0 – 1.404) |
| Thalamus iron deposits | 5 / 358 | 3 / 316 | 8 / 674 | 0.116 | 0.048 | 0.116 (0 – 0.116) |
| **Brain region** | **Number of microbleeds per region (*) in the sample** | | | **Frequency of occurrence (1 / more than 1 microbleed) in the sample** | | |
| Basal ganglia | 15 | 7 | 22 | 6 / 2 | 5 / 1 | 11 / 3 |
| Brainstem | 2 | 2 | 4 | 2 / 0 | 0 / 1 | 2 / 1 |
| Cortex / junction white matter | 48 | 12 | 60 | 9 / 6 | 5 / 2 | 14 / 8 |
| Deep white matter | 3 | 6 | 9 | 3 / 0 | 1 / 2 | 4 / 2 |
| Internal / external capsules | 2 | 1 | 3 | 2 / 0 | 1 / 0 | 3 / 0 |
| Thalamus | 2 | 1 | 3 | 2 / 0 | 1 / 0 | 3 / 0 |

Legend: BS iron deposits: brainstem iron deposits, WM iron deposits: white matter iron deposits, WMH: white matter hyperintensities, ICV: intracranial volume

(*) Data only refer to microbleeds visually identified as “certain” using BOMBS (Cordonnier et al., 2007)

*Table S2.* P-values resultant from the Mann-Whitney U test to explore the regional brain iron load (i.e. volume of iron deposits (IDs), in relation to gender and in the presence vs. absence of the vascular risk factors.

| **Parameters** | **Gender** | **Hyper-tension** | **Diabetes** | **Hyper-choleste-rolaemia** | **History of cardiovas-cular disease** | **History of stroke** |
| --- | --- | --- | --- | --- | --- | --- |
| **Volume of IDs in corpus striatum** | 0.593 | 0.280 | 0.067 | **0.002** | 0.821 | **0.006** |
| **Volume of IDs in brainstem** | **0.002** | 0.721 | **0.016** | 0.323 | **0.036** | **0.012** |
| **Volume of IDs in the cortex** | 0.153 (*) | 0.885 | 0.948 | 0.160 | 0.318 | **0.006** |
| **Volume of IDs in the white matter** | 0.821 | 0.218 | 0.938 | 0.135 | 0.874 | **< 0.001** |
| **Volume of IDs in the thalamus** | 0.460 | 0.529 | 0.203 | 0.116 | 0.278 | **0.015** |
| **Volume of IDs in the cortex, white matter, and thalamus together** | 0.871 | 0.306 | 0.722 | **0.029** | 0.991 | **< 0.001** |
| **Total volume of IDs** | 0.254 | 0.138 | **0.037** | **0.007** | 0.609 | **< 0.001** |

Note: “history of stroke” refers to a self-reported event of previous stroke/transit ischaemic attack or presence of an old stroke lesion detected on MRI. All volumes of regional iron deposits were corrected by head size (i.e. ICV). Correcting by brain size (i.e. brain tissue volume) yielded the same results.

(*) This value was the only one that differed (p=0.002) before adjusting for brain size.

*Table S3.* Linear regression results predicting the total volume of brain iron deposits in Model 1 (including fluid intelligence), Model 2 (including speed), and Model 3 (including memory). Bolded coefficients were statistically significant at *p* < 0.05.

| Predictor | Model 1  Total iron deposits  (*n* = 667) | | | | Model 2  Total iron deposits  (*n* = 659) | | | | Model 3  Total iron deposits  (*n* = 654) | | | |
| --- | --- | --- | --- | --- | --- | --- | --- | --- | --- | --- | --- | --- |
|  | *β* | SE | *p* | Adj. *R^2^* | *β* | SE | *p* | Adj. *R^2^* | *β* | SE | *p* | Adj. *R^2^* |
| Age | −0.075 | 0.039 | 0.054 |  | −0.065 | 0.039 | 0.099 |  | −**0.042** | **0.039** | **0.041** |  |
| Sex | −0.099 | 0.078 | 0.208 |  | −0.110 | 0.040 | 0.016 |  | 0.067 | 0.079 | 0.393 |  |
| Fluid intelligence | **−0.137** | **0.039** | **<0.001** |  | - | - | - |  | - | - | - |  |
| Speed | - | - | - |  | **−0.109** | **0.040** | **0.006** |  | - | - | - |  |
| Memory | - | - | - |  | - | - | - |  | **−0.110** | **0.039** | **0.005** |  |
| Hypertension | −0.012 | 0.081 | 0.879 |  | 0.025 | 0.082 | 0.755 |  | 0.013 | 0.082 | 0.878 |  |
| Diabetes | 0.109 | 0.130 | 0.399 |  | 0.128 | 0.130 | 0.324 |  | 0.144 | 0.132 | 0.276 |  |
| Hypercholesterolaemia | 0.158 | 0.083 | 0.059 |  | 0.143 | 0.084 | 0.088 |  | **0.175** | **0.084** | **0.038** |  |
| Cardiovascular disease | −0.029 | 0.090 | 0.756 |  | −0.055 | 0.090 | 0.540 |  | −0.019 | 0.090 | 0.831 |  |
| Stroke | **0.286** | **0.103** | **0.006** |  | **0.260** | **0.103** | **0.012** |  | **0.304** | **0.103** | **0.003** |  |
|  |  |  |  | 0.037 |  |  |  | 0.030 |  |  |  | 0.037 |
